# Antiretroviral Therapy and Risk of Stroke in People with HIV in Zambia

**DOI:** 10.21203/rs.3.rs-6945551/v1

**Published:** 2025-07-01

**Authors:** Stanley ZIMBA, Owen NGALAMIKA, Emmanuel MUKAMBO, Theresa SHANKANGA, Taonga MSIMUKO, Diwell MWANSA, Bwalya MULENGA, Mike CHISHA, Mashina CHOMBA, Melody ASUKILE, Lorraine CHISHIMBA, Violet KAYAMBA, Lloyd MULENGA, Omar SIDDIQI, Owen A. ROSS, Masharip ATADZHANOV, Deanna SAYLOR

**Affiliations:** University Teaching Hospital; University of Zambia School of Medicine; University Teaching Hospital; University Teaching Hospital; University Teaching Hospital; University Teaching Hospital; University Teaching Hospital; University Teaching Hospital; University of Zambia School of Medicine; University Teaching Hospital; University Teaching Hospital; University of Zambia School of Medicine; University Teaching Hospital; Beth Israel Deaconess Medical Center, Harvard Medical School; Mayo Clinic College of Medicine; University Teaching Hospital; University Teaching Hospital

**Keywords:** Antiretroviral therapy, HIV infection, stroke, Sub-Saharan Africa, Zambia

## Abstract

**Background:**

People with HIV (PWH) are at increased risk of stroke likely due to many factors including antiretroviral therapy (ART). We sought to evaluate the association between ARTand risk of stroke in PWH.

**Methods:**

We conducted a prospective case-control study at the University Teaching Hospital in Lusaka, Zambia between March 2022 and October 2024 in PWH comparing those with stroke (cases) and without (controls) matched (1:2) for age, sex and race. Standardized data collection instruments were used to collect demographic, clinical, laboratory and imaging information. Comparisons were made between the cases and controls, and subgroup analysis by ART duration was done for the cases.

**Results:**

We analyzed results for 205 cases and 410 controls. Compared to controls, cases were more likely to have hypertension (71% vs. 18%, *p*=0.001), lower CD4 counts [293(163–592) cells/μl vs. 533 (376–688) cells/μl, *p*=0.0001] and to be on second line ART (23% vs. 4%, *p*=0.001). Hypertension (aOR 19.7, 95% CI 3.1–126.4, *p*=0.002) and Tenofovir Disoproxil Fumarate (TDF) use (aOR 85.3, 95% CI 5.3–1380.7, *p*=0.002) were associated with increased odds of stroke, whereas Dolutegravir (aOR 0.03, 95% CI 0.001–0.58, *p*=0.02) and alcohol use (aOR 0.24, 95% CI 0.06–0.95) were associated with reduced odds of stroke. The majority of stroke patients on long-term ART were using Dolutegravir (80% vs. 35%, *p*=0.001) and TDF (72% vs. 42%, *p*=0.01).

**Conclusion:**

In PWH, TDF associates with higher odds of stroke. Although Dolutegravir associates with reduced odds of stroke, stroke patients on long-term ART are more likely to be on it.

## 1. Introduction

Sub-Saharan Africa (SSA) shoulders a disproportionate burden of stroke and HIV.^1–3^ These two epidemics are often intertwined and affect young adults aged less than 50 years without traditional stroke risk factors.^4, 5^ This presents a socioeconomic and public health crisis in SSA as the most affected individuals are productive members of society who are expected to propel economic development. HIV is increasingly recognized as an independent stroke risk factor.^6–8^ The exact mechanisms by which HIV increases the risk of stroke are not well-defined. Most of what is known about HIV-associated stroke comes from high-income settings where traditional stroke risk factors such as diabetes, hypertension, hyperlipidemia and smoking are more common.

HIV has an effect on vascular biology leading to an increased risk of cardiovascular and cerebrovascular diseases. This may occur as HIV-associated vasculopathy, indirectly through opportunistic infections,^9, 10^ or as a consequence of antiretroviral therapy (ART) drugs. Nucleoside reverse-transcriptase inhibitors such as Didanosine and Abacavir have been associated with increased cardiovascular risk whereas Tenofovir Disoproxil Fumarate (TDF) has consistently not demonstrated any such association in multiple cohort studies.^11–13^ Boosted protease inhibitors (PIs), which are the cornerstone of second line ART in many SSA countries such as Zambia, are associated with an increased risk of metabolic syndromes with longer exposure, which may worsen an underlying atherosclerotic process.^14^ All these drugs could increase the risk of cerebrovascular disease and stroke, but evidence linking them with such an association is lacking especially in SSA which is most affected by the HIV pandemic. Sub-Saharan Africa has a diverse and genetically different population which requires further investigation of ART and its contribution to stroke risk. Additionally, integrase inhibitors which form the backbone of first line ART in most African countries could potentially increase the risk of stroke within six months of ART initiation, as they rapidly suppress HIV which may possibly lead to an immune reconstitution inflammatory syndrome (IRIS)-like process.^10, 15, 16^

While the relationship between HIV and stroke has been explored, the specific role of ART – particularly with the widespread adoption of Dolutegravir (DTG) by World Health Organization (WHO) as the first-line ART backbone since 2016 – remains poorly understood.^17–19^ This matched case-control study aimed to investigate the association between ART, duration of use, and stroke risk among people with HIV (PWH). We hypothesized that DTG-based regimens would be associated with increased stroke risk within one year (recent) of ART initiation due to DTG’s rapid virological suppression and potential for IRIS stroke. We also hypothesized that PIs would be associated with increased stroke risk with long-term (≥ 1 year) use due to their increased risk for metabolic syndrome with long-term use.

## 2. Methods

### 2.1 Study Setting

The study was conducted at the University Teaching Hospital (UTH) in Lusaka, the largest and national referral tertiary care centre in Zambia. The hospital has neurodiagnostic assessments tools including magnetic resonance imaging (MRI), computed tomography (CT), and electroencephalography (EEG). The hospital has a neurology division with a functional stroke unit and a large adult centre for infectious diseases which attends to more than 20,000 PWH every year. Admission to UTH, physician consultations and medications stocked in the hospital pharmacy are free of charge for patients, but patients may pay out of pocket for all investigations if they do not have health insurance. As such, incomplete workups are common because of financial limitations or unavailability of reagents. In addition, investigations, which would not significantly alter patient management are usually not undertaken. However, UTH’s status as a national referral centre with advanced neurodiagnostic tools and a high volume of PWH makes it an ideal setting for investigating HIV-associated stroke.

### 2.2 Study Design and Period

We conducted a prospective case control study from March 2022 to October 2024 on PWH with (cases) and without (controls) stroke matched (1:2) for age, sex and race. Controls were consecutive patients accessing routine UTH outpatient HIV care services who were confirmed to be stroke-free after a neurological assessment. Cases were further stratified by ART use duration [recent (< 1 year) and long-term (≥ 1 year)].

### 2.3 Participants

All cases were adults (≥18 years) either admitted to the inpatient neurology service or seen at the neurology clinic at UTH with a clinical diagnosis of stroke in PWH on ART, with symptom onset of less than 2 months. All paper charts during the study period were reviewed to confirm the diagnosis of stroke. Of note, all patients admitted to UTH are routinely offered voluntary HIV testing and counseling upon admission. Patients with neuroimaging-confirmed ischemic stroke or intracerebral hemorrhage (ICH) were categorized accordingly; those seen with clinically suspected stroke who did not undergo neuroimaging due to financial constraints or scanner malfunction were categorized as “unknown stroke” (US). Exclusion criteria included transient ischemic attack (TIA) (symptom duration less than 24 hours), subdural or epidural hematoma, non-stroke neurological diagnoses, and stroke occurring secondary to surgery or traumatic injury.

### 2.4 Recruitment Process and Measurements

We leveraged the national electronic medical records for PWH (SMART Care), chart reviews and patient interviews to obtain data for cases and pre-enrolment data for controls including blood pressure, ART regimen and duration, CD4 count, and HIV viral load as well as socio-economic and clinical related factors. Routine stroke workup, including CT and MRI scans, lipid panel, electrocardiogram and echocardiogram, were also recorded if obtained as part of routine clinical care. All patients were assessed and examined by a neurologist (SZ). Stroke severity was graded according to the National Institutes of Health Stroke Scale (NIHSS) and the modified Rankin Scale (mRS).^20, 21^

Hypertension and diabetes mellitus were defined per World Health Organization (WHO) guidelines^22, 23^ and atrial fibrillation was defined by self-reported history or ECG or Echo confirming atrial fibrillation. Hyperlipidaemia was defined by self-report, statin use or standard laboratory cutoffs including low density lipoprotein (LDL) greater than 3.36 mmol/l, high-density lipoprotein (HDL) less than 1.29 mmol/l, total cholesterol more than 5.17 mmol/l or triglycerides greater than 3.88 mmol/l.^24^ Cigarette smoking was classified as active smoker (current or former smoker for less than 1 year), passive smoker (household member or coworker who regularly smoked in his/her presence for more than 1 year during the last 10 years) or nonsmoker, and alcohol intake (ex-drinker for less than 1 year or current alcohol intake).

### 2.5 Stroke Classification

A radiologist (MC) interpreted all brain imaging, and stroke classification was done independently by two study investigators (SZ and MA) with a third neurologist (DS) adjudicating when there was disagreement between the first two reviewers. Due to limited diagnostics, ischemic stroke cases were further classified using the Bamford classification: total anterior circulation infarction (TACI), partial anterior circulation infarction (PACI), posterior circulation infarction (POCI) and lacunar infarction (LACI).^25^

### 2.6 Sample Size

No pre-determined sample size was calculated for this exploratory study, but consecutive sampling was continued for a pre-defined period of one year. Based on our clinical registry,^5^ we had estimated to recruit 200 PWH with stroke during this recruitment period. The recruitment period was extended from one to two years due to disruptions caused by the COVID-19 pandemic.

### 2.7 Statistical Analysis

All data were entered into a secure REDCap database hosted by the Zambian Ministry of Health Infectious Diseases Directorate and analysed using SPSS version 27.^26, 27^ Demographic and clinical characteristics were summarized with descriptive statistics. Chi square test was used to determine the association between categorical variables. Student t-test was used to compare normally-distributed continuous variables between cases and controls, while the Mann-Whitney U-test was used if the continuous variables were not normally-distributed. *P* values of less than 0.05 were taken as statistically significant.

A multivariable conditional logistic regression model was then used to adjust for confounders and to identify factors independently associated with stroke in PWH. Only variables statistically significant at bivariate analysis were included in the multivariable conditional logistic regression model. Different models were run and the best predictive model was selected based on one with the largest area under the Receiver-operating characteristic (ROC) curve, lowest Akaike information criterion (AIC) and Bayesian information criterion (BIC), and the highest likelihood ratio. We checked fitness of the model using Hosmer-Lame-Show goodness of fit test and confirmed that there was no multicollinearity using variance inflation factor (VIF) values.

### 2.8 Ethical Approval

Ethical approval was obtained from the University of Zambia Biomedical Research Ethics Committee (No. 1945–2021) and National Health Research Authority (REF: NHREB00008/30/09/2021), while permission was obtained from UTH management to conduct the study at UTH. Written informed consent to participate in the study as well as for publication was obtained from each of the study participants. For participants with altered mental status, informed consent was obtained from a surrogate, defined as a caregiver or close relative.

## 3. Results

### 3.1 Demographics

**Figure 1:**
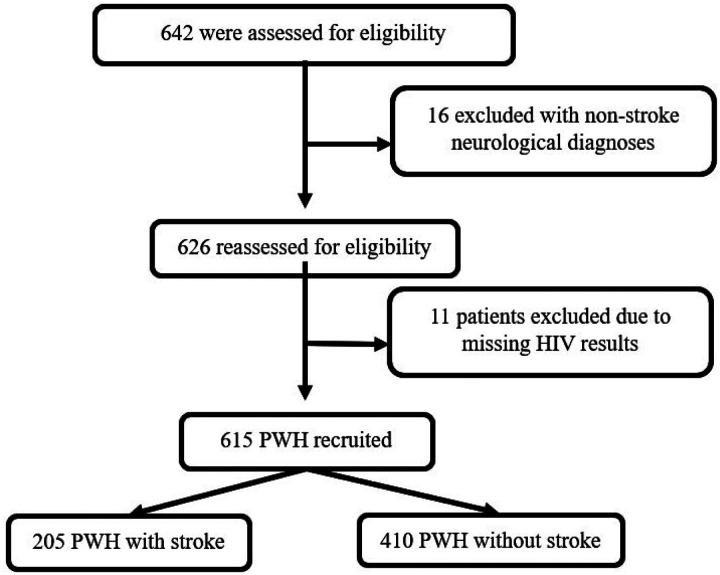
Recruitment process

In total, 205 cases and 410 controls were enrolled (Figure 1). The cases presented with a median NIHSS score of 9 (5–16) and mRS score of 4 (2–5). Ninety-one percent of the cases were identified as either ischemic or intracerebral hemorrhage and the rest were unknown as they did not get neuroimaging. For ischemic stroke, the majority (67%) of cases had anterior circulation infarction followed by lacunar infarction using Bamford classification (Table 1).

### 3.2 Risk Factors and Comorbidities

Compared to controls, cases were more likely to have traditional risk factors for stroke such as hypertension, diabetes, hyperlipidemia and atrial fibrillation. They also had lower CD4 counts and advanced WHO HIV clinical stage. Although 87% of cases were on long-term ART, they were significantly less compared to controls, and they were more likely to be on second line ART (Table 1).

### 3.3 Independent Predictors of stroke

Hypertension (aOR 19.7, 95% CI 3.1–126.4, *p*=0.002) and Tenofovir Disoproxil Fumarate (TDF) use (aOR 85.3, 95% CI 5.3–1380.7, *p*=0.002) were independently associated with increased odds of stroke, while Dolutegravir (DTG) use (aOR 0.03, 95% CI 0.001–0.58, *p*=0.02) and alcohol intake (aOR 0.24, 95% CI 0.06–0.95) were associated with reduced odds of stroke (Table 2). The prediction model used had area under ROC curve of 82% (Supplemental Figure 1).

### 3.4 PWH with Stroke and ART Use Duration

Of the 200 PWH and stroke taking ART, 174 (87%) were on long-term ART use. Those on long-term ART were less likely to be on second line ART, but more likely to be on TDF and DTG compared with their counterparts with recent ART use. Additionally, they were less likely to be on PIs compared with recent ART use, but the difference was not statistically significant (Table 3).

## 4. Discussion

This case-control study showed that stroke risk in PWH is associated with many factors including ART use. Notably, we found that TDF was associated with increased odds (aOR 85.3) of stroke in PWH. Protease inhibitors only showed increased odds of stroke at univariate analysis. Dolutegravir use was independently associated with reduced odds of stroke, but significantly more PWH and stroke were likely to be on DTG long-term use on subgroup analysis.

Our findings add a new dimension to the existing body of knowledge. For instance, TDF is known to be associated with better lipid profiles and potentially reduced cardiovascular risk, while its association with stroke risk has not previously been reported.^28^ Treatment with TDF has consistently been associated with lower lipid levels, a decline in renal function and reduced bone mineral density.^12, 29^ The mechanism by which it would be implicated in stroke risk is unclear, although its effect on renal function and its interaction with other ART drugs could provide a possible explanation for our findings, which need further research. In addition, the immunological and virological status of patients taking TDF may be important, as poorly-controlled HIV-infection can increase the risk of stroke.^8, 30^

As for DTG, it has been associated with increased risk of stroke within the first few months of its initiation due to its rapid virological suppression leading to a potential IRIS.^15, 16, 31, 32^ Our findings suggest an overall reduced odds of stroke with DTG use which could be explained by its beneficial effects on metabolic and inflammatory markers compared to other ART drugs.^33^ However, our findings showed that a higher proportion of long-term ART users with stroke were on DTG. This could suggest that long-term instead of recent DTG use is an important factor in stroke risk. Recent studies have demonstrated that DTG is associated with hypertension,^34^ hence its role in increasing stroke risk could also be indirectly through hypertension with prolonged use.

We found a modest association between boosted protease inhibitors (PIs) and increased odds of stroke which was not sustained on multivariable analysis. PIs are used in second line ART, and have been shown to increase cardiovascular disease risk by promoting metabolic syndrome with prolonged use.^35^ Our modest findings can be explained by the small numbers of recruited participants on PIs. Even on subgroup analysis of the cases, we observed that PWH and stroke were less likely to be on long-term use of PIs, but the difference was not statistically significant compared. It is important to emphasize that long-term ART use for our study was defined as 12 months or more whereas other studies which have looked at prolonged PIs use have considered more than 24 months.^36^ This timeframe difference could also have a bearing on our findings.

What is clear from our results is that both traditional and HIV-associated factors are important risk factors of stroke in this population of PWH. Hypertension was an independent risk factor of stroke in our study, with almost 20 times higher odds of stroke. However, we found the opposite for alcohol intake as it was noted to reduce the odds of stroke, although we did not properly quantify its use in our cohort. Several studies have shown that alcohol intake is an important risk factor for stroke with a direct relationship depending on quantities consumed, with some studies suggesting that alcohol intake in moderation is actually protective.^37–39^ Our findings ultimately support the current consensus that the actual mechanism of stroke in PWH is multifactorial, and ART may be a co-factor.

Our study has several limitations, including being a single-centre study at a national referral hospital, which might make the generalizability of these results difficult. As this was an exploratory study, we did not correct for multiple comparisons so that some of the associations found here may be due to chance alone. We did not thoroughly assess the contribution of the other ART drugs such as Abacavir, Zidovudine, Emtricitabine and Tenofovir Alafenamide which some of the participants may have been taking. Similarly, we did not compare for multiple ART combinations (e.g. some on the participants were taking both TDF and DTG) or the influence of the previous ART regimen for those who were recruited on second line ART. Nevertheless, our expectation is that these results are likely generalizable and they provide preliminary data that can be used to guide the development of interventions to reduce stroke risk in PWH. Some findings were based on information from the routine care available at the hospital during the study. The BMI for a few patients could not be calculated because of lack of a proper weighing scale for bedridden patients. Resource limitations also meant that not every participant was thoroughly evaluated for stroke risk factors such as hyperlipidaemia and diabetes (Supplemental Table 1). However, a strength of this study is the relatively large sample size and that all participants received a thorough clinical evaluation by trained neurologists making the clinical characterizations of this cohort more reliable than in many other SSA populations in which participants are assessed by non-neurologist healthcare workers.

In conclusion, PWH are at increased risk of stroke due to multiple factors including traditional risk factors such as hypertension and HIV-associated factors such as ART use. Tenofovir Disoproxil Fumarate increases the odds of stroke. Although Dolutegravir associates with reduced odds of stroke, stroke patients on long-term ART are more likely to be on it. We found a modest association between PIs use and increased stroke risk. Future research designed to focus on multiple combinations of the different ART drugs and their contribution to stroke risk is needed to provide better insight into these findings in order to allow for the development of targeted and effective interventions for both primary and secondary stroke prevention for PWH.

## Supplementary Files

This is a list of supplementary files associated with this preprint. Click to download.

- SupplementarymaterialAIDSResearch.doc

## Figures and Tables

**Table 1:** Comparison of demographic, clinical characteristics and ART factors between PWH with and without stroke

Characteristics	Cases (n=205)	Controls (n=410)	p-value
**Demographic/Clinical**			
Female n (%)	114 (56)	229 (56)	0.95
Age, mean (SD)	52 (±12)	52(±12)	0.95
Secondary school or higher n (%)	136 (66)	278 (68)	0.72
Married/cohabiting n (%)	107 (52)	212 (52)	0.91
Alcohol intake n (%)	59 (29)	264 (64)	**0.001**
Smoking n (%)	25 (12)	20 (5)	**0.001**
BMI (Kg/m^2^), median (IQR)	26.7 (22.9–32.2)	26.1 (23.5–29.2)	0.74
Systolic BP, mean (SD)	139(±29)	135(±13)	**0.04**
Diastolic BP, mean (SD)	89(±17)	79(±13)	**0.0001**
NIHSS, median (IQR)	9 (5–16)	NA	
mRS, median (IQR)	4 (2–5)	NA	
**Stroke Type**			
Stroke type n (%)		NA	
Ischemic	129 (63)		
Intracerebral hemorrhage	58 (28)		
Unknown stroke	18 (9)		
^¥^Bamford Classification of Ischemic Strokes, n (%):		NA	
LACI			
PACI	51/129 (39)		
TACI	36/129 (28)		
POCI	28/129 (22)		
	14/129 (11)		
**Comorbid Conditions**			
Hypertension n (%)	145 (71)	73 (18)	**0.001**
Diabetes mellitus n (%)	24 (12)	15 (4)	**0.001**
Hyperlipidemia n (%)	37 (18)	1 (0)	**0.001**
Previous stroke n (%)	56 (27)	NA	
Atrial fibrillation n (%)	5 (2)	1 (0)	**0.01**
**HIV-associated Factors**			
CD4 count (cells/μl), median (IQR)	293 (163–592)	533 (376–688)	**0.0001**
CD4 count ≥200cells/μl, n (%)	53/79 (67)	311/334 (93)	**0.001**
HIV viral load (copies/ml), median (IQR)	20 (0–241)	0 (0–29)	0.13
ART use, n (%)	200 (98)	404 (99)	0.39
ART duration, ≥ 1 year, n (%)	174/200 (87)	394/404 (99)	**0.001**
Second line ART n (%)	46/200 (23)	17/404 (4)	**0.001**
Dolutegravir n (%)	148/200 (74)	389/404 (95)	**0.001**
Tenofovir Disoproxil Fumarate n (%)	136/200 (68)	119/404 (29)	**0.001**
Protease Inhibitors n (%)	14/200 (7)	10/404 (2)	**0.01**
WHO HIV Clinical Stage n (%)			**0.001**
Stage 1	132 (64)	380 (93)	
Stage 2	37 (18)	21 (5)	
Stage 3	20 (10)	9 (2)	
Stage 4	16 (8)	0 (0)	

Bolded text signifies p < 0.05.

¥ Bamford classification pertains to those with confirmed ischemic strokes only, not the total sample

**Abbreviations:** ART=antiretroviral therapy; BMI=body mass index; BP=blood pressure; LACI=lacunar anterior circulatory infarct; mRS=modified Rankin Scale; NIHSS=national institute of health stroke scale; PACI=partial anterior circulatory infarct; POCI=posterior circulatory infarct; TACI=total anterior circulatory infarct; WHO=world health organization

**Table 2:** Multivariable conditional logistic regression analysis of factors associated with stroke in PWH

Variables	Adjusted OR (95% CI)	p–value (Adj. OR)
Alcohol intake n (%)	0.24 (0.06–0.95)	**0.04**
Hypertension n (%)	19.7 (3.1–126.4)	**0.01**
CD4 count (cells/μl), median (IQR)	1.000 (0.996–1.001)	0.24
Dolutegravir n (%)	0.03 (0.001–0.58)	**0.02**
Tenofovir Disoproxil Fumarate n (%)	85.3 (5.3–1380.7)	**0.01**
Second line ART n (%)	17.7 (0.9–353.9)	0.06

**Table 3.** PWH presenting with stroke stratified by duration of ART use

Characteristics	ART ≥ 1 year (n=174)	ART < 1 year (n=26)	p-value
Second line ART n (%)	31 (18)	15 (58)	**0.001**
Dolutegravir n (%)	139 (80)	9 (35)	**0.001**
Tenofovir Disoproxil Fumarate n (%)	125 (72)	11 (42)	**0.01**
Protease Inhibitors n (%)	11 (6)	3 (12)	0.40
